# Evaluation of pollinator effectiveness based on pollen deposition and seed production in a gynodieocious alpine plant, *Cyananthus delavayi*

**DOI:** 10.1002/ece3.3391

**Published:** 2017-09-05

**Authors:** Hao Wang, Guo‐Xing Cao, Lin‐Lin Wang, Yong‐Ping Yang, Zhi‐Qiang Zhang, Yuan‐Wen Duan

**Affiliations:** ^1^ Key Laboratory for Plant Diversity and Biogeography of East Asia Kunming Institute of Botany Chinese Academy of Sciences Kunming China; ^2^ Department of Forestry Sichuan Agricultural University Chengdu China; ^3^ Laboratory of Ecology and Evolutionary Biology State Key Laboratory for Conservation and Utilization of Bio‐Resources in Yunnan Yunnan University Kunming China

**Keywords:** gynodioecy, heterospecific pollen, pollen deposition, pollination efficiency, seed set

## Abstract

Examining variations in pollinator effectiveness can enhance our understanding of how pollinators and plants interact. Pollen deposition and seed production after a single visit by a pollinator are often used to estimate pollinator effectiveness. However, seed production is not always directly related to pollen deposition because not all pollen grains that are deposited on a stigma are compatible or conspecific. In the field, we tested pollinator effectiveness based on pollen deposition and the resulting seed production after single visits by different pollinator groups in a gynodieocious alpine plant *Cyananthus delavayi* (*Campanulaceae*). Our results showed that mean pollen deposition was generally inconsistent with mean seed production when comparisons were performed among different pollinator groups and sexes. In general, the correlations were not significant between pollen deposition and seed production in both perfect and female flowers after single visits by halictid bees, bumble bees, and hoverflies. We suggest seed set of virgin flowers after single visits is a more reliable indicator of pollinator effectiveness than pollen deposition and would be a better indicator of pollinator effectiveness for future studies.

## 1 INTRODUCTION

Plant‐pollinator interactions are one of the most pervasive mutualisms in nature as most flowering plants rely, at least in part, on insects or animals for reproduction. It has been well documented that not all floral visitors are pollinators and not all pollinators are equally effective in their pollination activities. For example, one pollinator species may visit multiple plant species and one plant species may also receive multiple pollinator species (Herrera, 1987, 2000; Wilson & Thomson, 1991). Differences in morphology and behavior may drive differences in pollinator effectiveness (Herrera, 1987; Primack & Silander, 1975; Solis‐Montero & Vallejo‐Marin, 2017). Thus, it is important to understand which visitors lead to reproductive success of plant species and assess the pollination performance of visitors to in turn understand the ecology and evolution of flowers (Ballantyne, Baldock, & Willmer, 2015; Dieringer, 1992; King, Ballantyne, & Willmer, 2013; Olsen, 1996; Sahli & Conner, 2007).

An effective pollinator is one that deposits sufficient conspecific pollen to receptive stigma at the right time. Pollinator effectiveness, the contribution of a pollinator to plant fitness, is often used to rank the importance of different species of visitors as pollinators (Gross, 2005; Ne'eman, Jurgens, Newstromlloyd, Potts, & Dafni, 2010; Primack & Silander, 1975). There is a long research tradition of seeking to improve our understanding of pollinator effectiveness (e.g., Adler & Irwin, 2006; Ne'eman et al., 2010; Primack & Silander, 1975; Wilson & Thomson, 1991). Multiple methods of field experiments have been developed to examine the pollinator effectiveness of a given plant species (Ne'eman et al., 2010). Single‐visit pollen deposition measures both a visitor's ability to acquire pollen in earlier visits to the plant species and the accuracy of deposition so it results in successful fertilization (Ne'eman et al., 2010). Practically, the number of pollen grains deposited on stigma is often considered to be a measure of pollinator effectiveness because it is easily obtained in the field, and there are ecological links between the two factors. Recent studies have demonstrated that single‐visit pollen deposition on virgin stigma is a practical measure of pollinator effectiveness (Ballantyne et al., 2015; King et al., 2013; Willmer, Cunnold, & Ballantyne, 2017). However, single‐visit pollen deposition cannot accurately represent final female reproductive success of a flower. First, it is difficult to distinguish between conspecific and heterospecific pollen grains. Pollen loads on stigma might not be related to potential seed production due to heterospecific pollen transfer (Benjamin & Winfree, 2014; Galen & Gregory, 1989; Morales & Traveset, 2008). Second, the pollen number on stigma as a measure of pollination effectiveness does not consider the fate of pollen on the stigma. For example, some deposited conspecific pollen grains may not germinate, especially if large numbers clog up a small stigma. Therefore, some studies have estimated pollinator effectiveness directly as seed set or fruit set resulting from a single visit by each taxon‐visiting plants (Ne'eman et al., 2010). However, using seed production to assess pollinator effectiveness also requires some additional considerations. For example, insufficient resources may reduce seed set as available resources often limit female fitness (Bateman, 1948). To improve our understanding of pollination ecosystem services and plant‐pollinator interactions, a clarification of the relationship between the number of pollen grains deposited on a stigma and the resulting seed set after single visit is needed.

Here, we investigate different insect pollinator effectiveness in hermaphrodite and female individuals of the gynodioecious plant *Cyananthus delavayi* by comparing the number of pollen grains and seed production of the same virgin flowers after single visits. This species exists in the highly diverse vegetation communities of southwest China where heterospecific pollen deposition is common (Fang & Huang, 2013). Pollinator effectiveness could affect the seed set in hermaphrodite and female individuals and consequently the maintenance of gynodioecy in natural populations. The objectives of this study were to (i) evaluate pollinator effectiveness based on pollen deposition and seed set after single visits by different pollinator groups, and (ii) examine the relationships between pollen deposition and seed set after single visits.

## 2 MATERIALS AND METHODS

### 2.1 Study species and site

*Cyananthus delavayi* Franch. (*Campanulaceae*) is a prostrate herbaceous perennial endemic to the alpine area of southwest China (Hong & Ma, 1991). It produces showy violet–blue tubula campanulate flowers, with five corolla lobes. The flowering season is usually from mid‐August to late September. The number of flowers produced per plant ranges from dozens to hundreds depending on the age of the plant. The target population of *C. delavayi* is gynodioecious, and thus we conducted the experiment in females and hermaphrodites. Halictid bees (*Halictus* sp.), bumblebees (*Bombus richardis* and *B. festivus*), and hoverflies are the main visitors to *C. delavayi* (Niu, Yang, Zhang, Li, & Sun, 2011). We used bumblebees, halictid bees, and hoverflies as the different pollinator groups to compare the number of pollen grains on stigma and seed production.

This study was conducted at Shangri‐La Alpine Botanical Garden (27°54′5″N, 99°38′17″E, 3300–3350 m above sea level), Yunnan Province, southwest China, in 2016. In the study population, flowers of *Pedicularis* species were common in the meadow, and *Astragalus pullus*,* Halenia elliptica*, and some *Apiaceae* species were less abundant (Fang & Huang, 2012).

### 2.2 Pollen deposition and seed production

In order to measure single‐visit pollen deposition, we randomly selected 30 female plants and 30 hermaphrodite plants in full‐bloom during the growing season of 2016. We removed all opened flowers on the target plants and excluded pollinators by placing nylon nets over the plants. When flowers opened, they were individually unbagged and observed until each received one insect visit. Visitor type was recorded, and the flowers were then tagged and rebagged. A previous study showed that a higher proportion ovules were fertilized 4–7 hr after pollination (Niu et al., 2011). As such we collected the stigmas of marked flowers 8–10 hr after visitation and stored them separately in 70% ethanol in a 1.5‐ml microcentrifuge tube. In the laboratory, we counted pollen grains on each stigma. The stigmas were softened in 8 mol/L NaOH solution for 8 hr and then dyed with an aniline blue solution (1%) after rehydration in distilled water for 2 hr. The stigma was then flattened on a slide. Pollen that dropped off the stigma during storage was collected by centrifugation and the resulting 30 μl of solution was then transferred to a slide. All pollen grains on each slide were counted using a compound binocular microscope at 40× magnification. Unvisited flowers were also netted as controls and pollen grains on their stigmas were recorded to account for self‐pollination by wind. All fruits of marked flowers were collected after 30 days, and the number of seeds was determined. Table 1 shows sample sizes that were used to analyze pollen deposition on the stigmas and seed set after single visits.

**Table 1:** Sample sizes of *Cyananthus delavayi* for single‐visit pollen deposition and seed set

Visitor	Flower sex	Sample size
Bumblebee	Hermaphrodite	23
	Female	32
Halictid bee	Hermaphrodite	20
	Female	19
Hoverfly	Hermaphrodite	25
	Female	28

To test the effects of pollinator group and plant sex on pollen deposition and seed production, multiple linear regression with a Poisson distribution error was used. The model included pollinator group (bumblebee, halictid bee, and hoverfly), sex (female and hermaphrodite), and their interaction as fixed effects. The significance of fixed effects was examined through *F* tests using analysis of variance (ANOVA). Because the sample sizes in this study were small (Table 1), a power analysis using *k* = 3, *f*‐level = 0.4, *p*‐level = .05, and power‐level = 0.8 was conducted. In this analysis, *k* is the number of pollinator group and *f* is the effect size. Pearson correlation was used to analyze the relationship between single‐visit pollen deposition and that of seeds per fruit for each pollinator group in both female and hermaphrodite flowers. All analyses were carried out using R (R Core Team, 2017, version 3.32).

## 3 RESULTS

**Figure 1:**
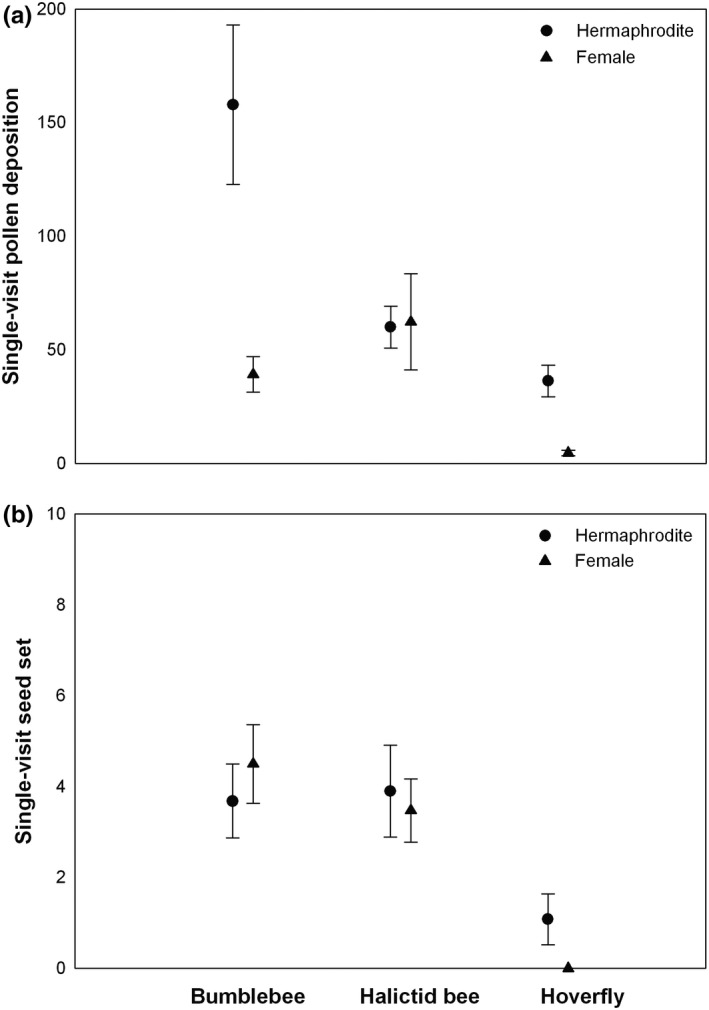
Pollen deposition (a) and seed production (b) after single visits by bumblebees, halictid bees, and hoverflies

Pollen deposition after single visits was significantly affected by plant sex, pollinator group, and their interaction (Table 2). Pollen grains deposited by bumblebees and hoverflies were significantly higher for hermaphroditic flowers than for female flowers (both *p* < .01), but pollen grains deposited by halictid bees did not differ significantly between hermaphrodites and females (*p* > .8; Figure 1a). The seed production of flowers after single visits was affected significantly by pollinator group, but not by plant sex and their interaction (Table 2). A similar number of seeds for hermaphroditic and female flowers were observed after single visits by pollinators (Table 3), and a similar number of seeds were observed with single visits by bumblebees and halictid bees (*p* > .7). Single visits by hoverflies resulted in significantly fewer seeds than single visits by bumblebees and halictid bees (both *p* < .01; Figure 1b).

**Table 2:** Results of the linear model to test the effects of pollinator group (bumblebees, halictid bees, and hoverflies) and sex (female and hermaphrodite) on pollen deposition and seed production in *Cyananthus delavayi*

Factor	*D.F*	Pollen deposition		Seed production	
		*F*	*p*	*F*	*p*
Sex	1	15.246	<0.001	0.069	.794
Pollinator group	2	10.455	<0.001	15.591	<.001
Sex × Pollinator group	2	6.758	<0.005	0.960	.385
Residuals	143				

**Table 3:** Results of the linear model to test the effects of pollinator group, sex, and pollen deposition on seed production in *Cyananthus delavayi*

Factor	*D.F*	*F*	*P*
Sex	1	0.067	.796
Pollinator group	2	15.231	<.001
Pollen deposition	1	0.036	.850
Sex × Pollinator group	2	1.072	.345
Sex × Pollen deposition	1	0.589	.444
Pollinator group × Pollen deposition	2	0.127	.880
Sex × Pollinator group × Pollen deposition	2	0.776	.462
Residuals	137		

**Figure 2:**
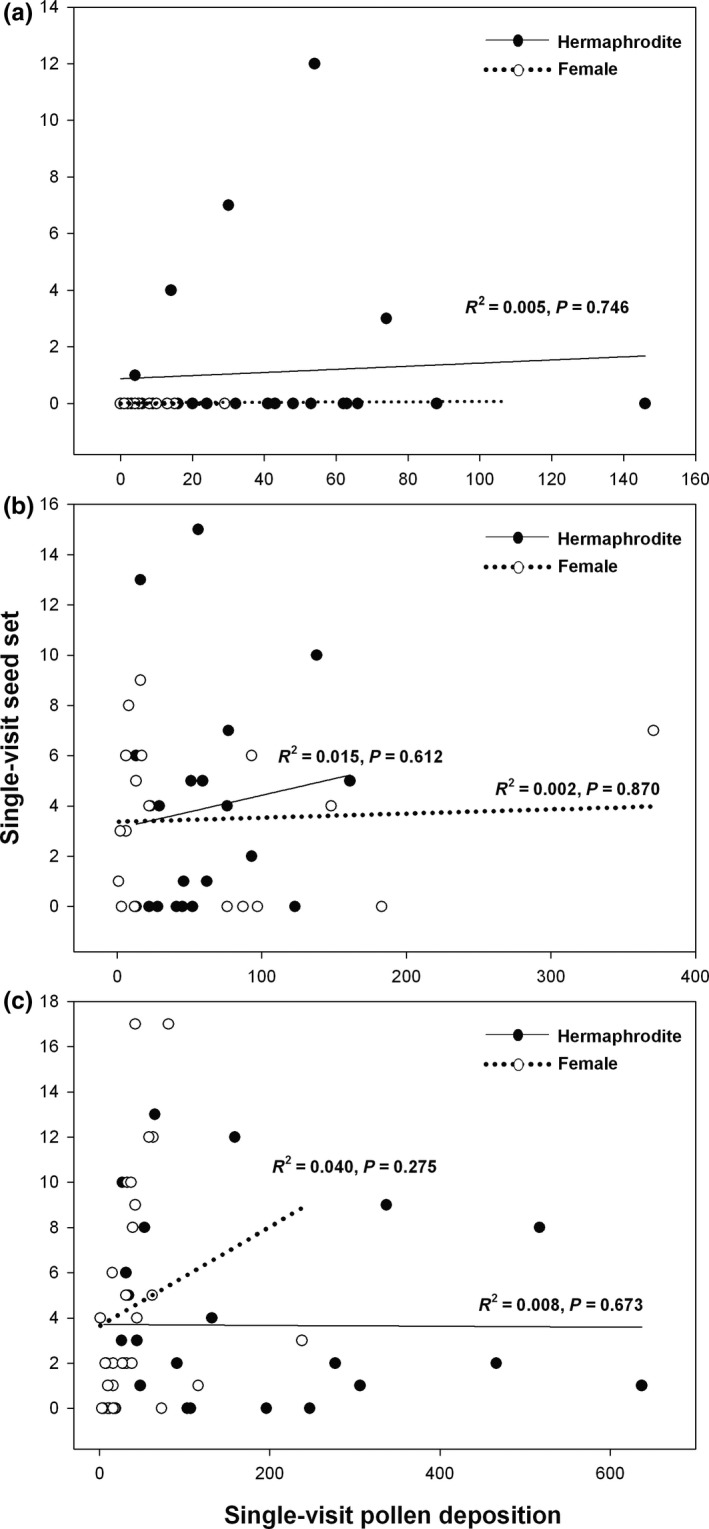
The relationship between pollen deposition and seed production of perfect and female flowers of *Cyananthus delavayi* after single visits by hoverflies (a), halictid bees (b), and bumble bees (c)

In flowers visited by hoverflies and halictid bees, there was no significant correlation between the number of pollen grains per stigma and seed number per fruit in hermaphrodite and female individuals (Figure 2). In flowers visited by bumblebees, there was no significant correlation between the number of pollen grains per stigma and seed number per fruit of perfect and female flowers (Figure 2c). According to the power analysis, the results can be regarded as reliable when the number of samples in each group is close to 21. In our study, the sample sizes in the three pollinator groups for hermaphrodite plants are 23, 20, and 25, respectively, and the sample sizes for female plants are 32, 19, and 28, respectively making the results acceptable.

## 4 DISCUSSION

Not all visitors to a given plant species are effective pollinators. To be an effective pollinator, conspecific pollen must be deposited on receptive stigma and result in successful seed set. Our results suggest that both bumblebees and halictid bees are effective pollinators of *C. delavayi* as set seed after single visits by both insects, which was less than that of hand‐pollinated flowers (Zhang Z.‐Q. and Wang H., unpublished data). Hoverflies appear to be less effective as almost no seeds were produced even though pollen grains were found on the stigma after single visits. The relatively short tongues of hoverflies compared with bees, combined with their behavior of making less bodily contact with sex organs and low visitation rates further decrease their importance as pollinators. Size matching between flowers and visitors has been suggested as a contributor to pollinator effectiveness (Solis‐Montero & Vallejo‐Marin, 2017). Interestingly, nectar‐feeding bumblebees and pollen‐collecting halictid bees, which are vastly different in size, had similar effectiveness, while halictid bees and pollen‐feeding hover flies, which are similar in size, had very different effectiveness. This suggests that visitor foraging behavior rather than size matching is an important factor in determining pollinator effectiveness.

Both pollen deposition on the stigma and seed set after single visits have been used previously to estimate pollinator effectiveness (Ne'eman et al., 2010). However, we observed weak correlations between pollen deposition and seed production. A possible explanation for this is the transfer of heterospecific pollen, which does not result in seed production. Flowering plants often share pollinators in natural communities (Bascompte, Jordano, Melián, & Olesen, 2003; Mitchell, Flanagan, Brown, Waser, & Karron, 2009; Nikolas, Waser, Chittka, Price, Williams, & Ollerton, 1996) and receive pollen from multiple heterospecifics as well as conspecifics. Heterospecific pollen transfer is common in nature (Galen & Gregory, 1989; Mitchell et al., 2009; Morales & Traveset, 2008; Nickolas M Waser & Fugate, 1986), and thus pollen grains that arrive on a stigma may not always be compatible or conspecific (Rathcke, 1983; N. Waser, 1983). Previous studies have indicated heterospecific pollen can interfere with conspecific pollen deposition and germination (Galen & Gregory, 1989), as well as with pollen tube growth, ovule fertilization, and seed development (Morales & Traveset, 2008; Wilcock & Neiland, 2002). Galen and Gregory (1989) found that the prior deposition of heterospecific pollen reduced the amount of conspecific pollen and subsequent germinating reducing the fertilization success by conspecific pollen grains in flowers of *Polemonium viscosum*. In our study site, bumblebees and halictid bees were found to pollinate many plant species (Fang & Huang, 2013), which could explain the weak relationship between pollen deposition and seed production after single visits. Additionally, because *C. delavayi* is self‐compatible (Niu et al., 2011), self‐pollination could occur in hermaphrodite flowers. The proportion of self‐ versus outcross‐pollen could also affect the relationship between pollen deposition and seed production and might partially explain the difference in bumblebee pollination of hermaphrodite versus female flowers in Figure 1a. It is possible that bumblebees, due to their large size, caused enough disturbance in hermaphrodite flowers that self‐pollination occurred in addition to the conspecific pollination. Self‐pollinated flowers might set fewer seeds than outcross‐pollination because of inbreeding depression cause higher rates of seed abortion during seed development (Montalvo, 1992). In addition, the weak correlation between pollen deposition and seed set could be due solely to small sample sizes and the variance within each group.

## 5 CONCLUSIONS

We found no significant correlations between pollen deposition on individual stigmas and seed set after single visits for the different pollinator groups for *C. delavayi*. This is likely caused by both heterospecific pollen deposition and the quality of pollen deposited. We conclude that variation in pollen deposition is and unreliable proxy for pollinator effectiveness, as it is weakly related to reproductive success. Our results suggest seed set of virgin flowers after single visit is an accurate, simple, and direct measure of pollinator effectiveness and should be assessed in more plant species in future studies.

## CONFLICT OF INTEREST

The authors declare that they have no competing interests.

## AUTHOR'S CONTRIBUTIONS

YWD and ZQZ conceived and designed the experiments. HW, GXC, LLW, and YYP performed the experiments. YWD, ZQZ, YPY, GXC, and HW analyzed the data and wrote the manuscript. All authors read and approved the final manuscript.
